# How to measure barriers in accessing mental healthcare? Psychometric evaluation of a screening tool in parents of children with intellectual and developmental disabilities

**DOI:** 10.1186/s12913-022-08762-0

**Published:** 2022-11-21

**Authors:** Ting Xiong, Elisa Kaltenbach, Igor Yakovenko, Jeanine Lebsack, Patrick J. McGrath

**Affiliations:** 1 University of Toronto, Toronto, Canada; 2 IWK Health Centre, 5980 University Ave #5850, Halifax, NS B3K 6R8 Canada; 3 Dalhousie University, Halifax, Canada; 4 Parent partner, Halifax, Canada

**Keywords:** Healthcare barriers, Healthcare access, Scale development, Scale validation, Parent, Caregiving, Intellectual and developmental disabilities

## Abstract

Caring for children with intellectual and developmental disabilities (IDD) can cause an enormous physical and emotional burden, and therefore these parents have an elevated risk to experience mental health problems. The characteristics of current healthcare systems and parents’ responsibilities to care for their children seem to impede their access to mental healthcare. There is so far a lack of instruments to screen for such obstacles. The aim of this study was to develop and validate a scale for measuring barriers to accessing mental healthcare. The Parental Healthcare Barriers Scale (PHBS) was developed on the basis of an extensive literature research, input and discussion from experts and parents with lived experience. A cross-sectional survey was used to collect data from 456 parents of children with IDD. Physical health, mental health, social support, and parenting were measured for concurrent and discriminant validity of the PHBS. The PHBS scale revealed acceptable to good reliability and validity. It consists of four subscales (i.e., support accessibility, personal belief, emotional readiness, and resource availability). The PHBS found parents prioritized their children’s treatments over their own mental health challenges (93.4%), did not have enough time (90.4%), and had financial concerns (85.8%). Parents in rural and remote areas had more limited resources. Findings from our study suggest increasing financial support for the parents seeking mental health services, introducing evidence-based treatments, increasing the availability of healthcare services for parents, and adjusting current services to their needs.

**Supplementary Information:**

The online version contains supplementary material available at 10.1186/s12913-022-08762-0.

## Background

Intellectual and Developmental Disabilities (IDD) often has an early onset and impedes one’s ‘intellectual, motor, language, or social functions’ chronically [1]. The existence of IDD and its relevant health outcomes affect the individual as well as their family. This is particularly salient for parents who care for children with IDD and see their children suffer. Considerable research shows that caring for children with IDD or other chronic diseases can cause physical and emotional burden [2–4].

Mental health disorders such as posttraumatic stress disorder (PTSD), depression, and anxiety are heightened in such parents [5–7]. Although little research summarizes the prevalence of such disorders in parents of children with a variety of IDDs, a PTSD prevalence of 10–30% was found in parents of children with epilepsy [5], autism spectrum disorder [8], and other chronic illnesses [9]. Considering the interrelated dynamics between parental well being, children’s health outcome, and familial support, it is vital to improve parents’ access to mental healthcare services [10].

However, a variety of factors may hinder parents’ access, availability and readiness to participate in treatments for their own mental health [11, 12]. A preliminary study from Australia [13] discovered that parents of children with intellectual disabilities reported costs, childcare arrangement, and mental healthcare availability as barriers for accessing mental health treatment. Bowling et al. [14] reported that lack of resources and support were main barriers confronted by parents of children with neurodevelopmental and mental disorders in the USA. The same barriers were found in families of people with autism spectrum disorder in Latin American countries [15]: the long waitlists, high service costs, and lack of access to treatment. To date, a systematic investigation of the various factors that impede parents of children with IDD from receiving mental healthcare services is not yet available.

Gulliford et al. [16] summarized general healthcare barriers as manifesting on personal, financial, and organizational levels and the theme of access was conceptualized in four dimensions: service availability/accessibility, acceptability, affordability, and accommodation. Although the Government of Canada and the provinces have devoted an increasing investment in mental healthcare [17, 18], the accessibility of services is still a major barrier. There was little updated evidence about the sufficiency of health services for parents of the neurodiverse children. For the general population, it was estimated that only 1 in 5 Canadians reported that their need for mental health services was met [19]. There is a widely reported shortage of mental healthcare providers, including psychologists, psychiatrists, social workers, and family physicians in Canada and across the world [20, 21]. This leads to long wait lists and wait time for mental health diagnoses and treatments [22]. In rural and remote areas services, there are even more scarce and challenging to the access. Despite the limited supply of services, the inequitable allocation of these services has caused the additional difficulty of accessing care in rural and remote areas [23, 24].

Moreover, the acceptability can further limit parental access to mental healthcare. Acceptance is connected to personal beliefs and previous experiences with mental health services [25]. For example, the stigma underling mental illness may discourage parents seek support [26]. Parents may also experience feelings of guilt about having mental health problems while caring for their children [27]. Other documented personal barriers are emotional readiness [28], confidentiality concerns [29], and concerns regarding the usefulness of treatments [30].

Affordability has been revealed as a key barrier to accessing mental healthcare for parents of neurodiverse children as well as the general population [15, 31]. Psychological services are not fully, if at all, covered by insurance [32]. Parents often prioritize spending on their children’s rather than on their own needs [33, 34].

Understanding the barriers parents face when accessing or trying to access mental healthcare is essential to facilitate decreasing these barriers. So far there is a lack of validated instruments to quantify barriers to access mental health services among parents affected by their children’s illnesses. This study aims to explore such barriers in the context of parenting a child with chronic illness like IDD, to develop and validate a scale for measuring barriers to access mental healthcare, and to examine the relationship between parental experienced barriers and their geographic setting (i.e., urban setting, suburban setting, and rural/remote setting).

## Method

### Participants and procedure

In total, 456 parents of children with an IDD participated in this study. This study was a part of a larger survey for these parents [35, 36]. They were recruited in Canada starting in 2020. The study was approved by the Research Ethics Board (REB) of a local hospital (IWK Health Centre REB# 1025477). The participants were recruited through online platforms (e.g., organizational websites, newsletters, personal blogs, and social media). Participants were directed to the Research Electronic Data Capture (REDCap [37];), where their data were collected and stored. Recruitment efforts were made by the research team and a Parent Advisory Committee, which included a group of parents of children with IDD. They assisted the study recruitment as well as the development of the survey.

The participants firstly clicked a link and read the consent form. After they consented, their eligibility was assessed by two questions that asked if they were: (1) parents (i.e., biological parents, foster parents, primary caregivers) of children with an IDD (any age); and (2) living in Canada. Ineligible participants were not invited to the PHBS survey. Following this, participants started the survey and were given option to enter into a gift card draw at the end. Three participants were randomly chosen for a $100 gift card per person.

### Measures

#### Barriers

This study followed the recommended best practices in scale development for health research [38], which includes three stages, namely item development (i.e., identifying domain, generating items, and assess content validity), scale development (i.e., question pre-testing, sampling and administering the scale, reducing items, and extracting factors), and scale evaluation (i.e., testing dimensionality, reliability, and validity).

The scope of the Parental Healthcare Barriers Scale (PHBS) was intended to be potential factors that negatively affect access to mental healthcare among parents of children with IDD. A literature search confirmed no existing instruments within this scope. Dimensions of the domain were not specified a priori as no consensus was yielded on this issue [39–41].

During the item development stage, literature on barriers of accessing healthcare and barrier scales developed for other traumatized or vulnerable populations (e.g., veterans) were searched. During the literature search, barriers perceived by parents of children with IDD and other comparative populations were identified. Some common obstacles were solicited: long wait lists, high costs [15], lack of resources [14], and personal beliefs [25]. A scale on barriers to treatment on children’s physical diseases included negative beliefs about treatments, and personal connection with healthcare deliverers. Other revealed themes were financial concerns, healthcare intervention and clinician availability, and stigma [40].

Following this, researchers (TX and JL), clinicians (EK and PJM), and parents of children with IDD (i.e., a group of 7 parent advisors and 5 parent ambassadors) discussed the development of items and evaluated face and content validity of the PHBS during the biweekly meetings in the 3-month development phase and 2 rounds of written feedback. Each provided independent comments and suggestions on the item comprehensiveness, clarity, relevance, and instructions and format of the scale. The recommendations were carefully considered for revision. The following changes were made: (1) item wording was revised to increase understandability; (2) instructions were slightly altered to match the scale domain; (3) response format was changed from dichotomous style to Likert scaling from 0 (not at all) to 4 (extremely); (4) an open-ended question (i.e., specification of other unlisted barriers) was added; (5) irrelevant items were deleted and items with overlapping meaning were merged. For instance, two items on treatment costs (i.e., “treatment expense is too expensive” and “other incidental costs are too high”) were combined into a single item (i.e., “the expense and added costs (e.g., time off work, transportation) are too high”). Finally, a 16-item PHBS was generated to use in this study with a total between 0 and 64; a higher score means more barriers were encountered by the respondent.

Due to the scarce quantitative study in parental barriers to seeking help for mental health problems [25, 26, 42, 43], there was no available instruments for testing convergent validity of the PHBS. As mental healthcare barrier is a broad concept with limited solid framework [44], the detection of strong correlates of PHBS was not available. From other quantitative studies on the barriers [39, 45, 46], social support [43], general physical health, and mental health [47] have been found moderately correlated with barriers to mental healthcare access, of which the lack of social support showed a slightly stronger correlation [45]. Therefore, they were evaluated in the current study for assessing concurrent validity. Moreover, parenting behaviors and parent-child interactions were assessed to evaluate the discriminant validity as they were found as an insignificant correlate of parental health-related behaviors and attitudes [48].

#### Physical health

Participants’ general physical health was assessed to calculate concurrent validity of the PHBS by the PROMIS Global Physical Health Scale (PROMIS GPH-4 [49];). This was conducted in line with the current evidence on the association between physical health problems and diminished help-seeking behavior [50, 51]. It consists of 4 items asking about the overall physical health, physical function, pain, and fatigue. The physical pain item was rated on a 0–10 scale (0 = no pain, 10 = worst pain imaginable) and the remaining 3 questions were rated on a 1–5 scale. A higher score indicates a worse health status. Hays et al. [49] reported that the internal consistency of the PROMIS GPH-4 was .81. In our sample, the Cronbach’s α was α = .655.

#### Mental health

Overall mental health was administered to evaluate the concurrent validity of the PHBS scale via the PROMIS Global Mental Health Scale (PROMIS GMH-4 [49];). It includes 4 items, assessing quality of life, mental health, satisfaction with social activities, and emotional problems. The GMH-4 was rated through a 5-point Likert scale ranging from 1 to 5 (range = 5–25). Higher scores indicate a worse mental health status. The scale showed high reliability and validity in primary care settings [52]. In this study, the internal consistency was Cronbach’s α = .822.

#### Social support

Perceived social support was measured to calculate the concurrent validity of the PHBS scale as it was found as a facilitator towards accessing mental healthcare [53]. The Multidimensional Scale of Perceived Social Support (MSPSS [54];) was utilized for this purpose. The 12-item scale assesses perceived support from family (4 items), friends (4 items), and significant others (4 items). Each item was rated from 1 to 7 and the range was 12–84. A total score was calculated with a higher level of social support represented by a higher sum score. In our study, the internal consistency for the MSPSS was Cronbach’s α = .922.

#### Parenting

To calculate the discriminant validity of the PHBS scale, the Parenting scale of the Parent and Family Adjustment Scales (PAFAS [55];) was used due to the heterogeneous nature of the two concepts [48]. A total of 18 items examine parenting in 4 dimensions, namely parental consistency (5 items), coercive parenting (5 items), positive encouragement (3 items), and parent-child relationship (5 items). One item (i.e., item 9) was deleted due to legal concerns about spanking children. The remaining 17 items were scored from 0 to 3 on a Likert scale; the total score ranges from 0 to 51. Higher scores indicate worse parenting, including lower consistency, more coercive parenting, less positive encouragement, and worse parent-child relationship. It has been validated for use with children with IDD [56]. The PAFAS-Parenting scale showed a Cronbach’s α of .715 in the current study.

### Data analysis

The collected data were analyzed with the software IBM SPSS Statistics 26 and R 4.0. In the scale development phase, item reduction and extraction of factors were conducted. The quantitative assessment was guided by the classical test theory model (CTT [57];) and the item response theory model (IRT [58];). In the scale evaluation phase, dimensionality, reliability, and validity were tested.

We first calculated the correlations between each PHBS item and the total score. Then an item discrimination index was estimated for each item to assess its ability to differentiate a high barrier and a low barrier group. For this, we used the 75th percentile total score and 25th percentile total score as two cut-off scores for the upper and lower group. The item discrimination index was calculated by the following function: Discrimination Index = P_*U*_ – P_*L*_; P_*U*_ = (number of cases endorsed the barrier in upper group/number of total cases of the upper group) × 100%; P_*L*_ = (number of cases endorsed the barrier in lower group/number of total cases of the lower group) × 100% [59].

To extract factors within parents’ barriers in accessing healthcare, a principal components analysis (PCA) was conducted as there was no existing theoretical model in the measured concept. The open-ended question (item 16 that asked other barriers beyond the listed items) was not included in the calculation of dimensionality. This is because the last open-ended question was not suitable for the factor extraction and an exploratory analysis showed that it did not appear in any component load (rotated component coefficients = .220, .055, .095, .113 for component 1, 2, 3, and 4, respectively). The applicability of the PCA analysis for the data set was assessed before the analysis. Inspection of the correlation matrix showed that all variables had at least one correlation coefficient greater than 0.3. The overall Kaiser-Meyer-Olkin (KMO) measure was .79 with individual KMO measures all greater than 0.6 classifications of ‘mediocre’ to ‘marvellous’ according to [60]. Barlett’s test of sphericity was statistically significant (*p* < .0005), indicating that the data were likely factorizable.

The internal consistency was estimated for both the total scale and the extracted factors of the PHBS. The concurrent validity was reported by its Spearman’s rank correlation coefficients with physical health (assessed by PROMIS GPH-4), mental health (assessed by PROMIS GMH-4), and social support (assessed by MSPSS) because all scales were scored on Likert style and the test of distribution of scores on PHBS violated assumption of normality, as assessed by Shapiro-Wilk’s test (*p* < .001). The discriminant validity was reported by its correlation with parenting (assessed by PAFAS-Parenting).

To compare the effect of geographical settings (urban/suburban/remote and rural) of parental barriers to mental health services, a one-way ANOVA was conducted to determine if the barrier levels (as calculated by the sum scores of the PHBS and its subscales) were different for the three geographic groups. The relative statistical assumptions for the one-way ANOVA were tested. There were no outliers, as assessed by boxplots; data was approximately normally distributed for each group, as assessed by histograms and Q-Q plots; and there was homogeneity of variances, as assessed by Levene’s test of homogeneity of variances (*p* = .901 for PHBS total score).

## Results

### Descriptive statistics

Demographic information of the study participants is presented in Table 1. Approximately 94.7% were female, with an average age of 43.12 years (*SD* = 7.58). Their children with an IDD were on average 11.63 years old (*SD* = 5.94). Most of the parents had a university degree (61.6%) and were married or in a domestic partnership relationship (76.9%). Nearly half were not employed or unpaid caregivers for their children. They spent on average 114.23 hours per week taking care of their children (*SD* = 52.39). A multiple linear regression model was run to explore whether demographic variables (i.e., parents’ age, children’s age, years of children’s IDD diagnoses, number of children the parent had, number of children with IDD the parents had, and weekly caregiving hours) predicted parental barriers of mental healthcare. Only numbers of children with IDD the parent had (Beta = .19, *p* < .01) and weekly caregiving hours (beta = .22, *p* < .001) were found as significant predictor of parental mental healthcare barriers.

**Table 1:** Sociodemographic Characteristics of the Study

Demographic Characteristics		***N***	***%***
**Sex**		**454**	
Female		430	94.7%
**Relationship**		**456**	
Biological Parent		414	91.0%
Adoptive Parent		35	7.7%
Step-parent/Legal Guardian		6	1.3%
**Level of Education**		**445**	
High School		49	11.0%
Occupational/Technical/Vocational Training Occupational/Technical/Vocational Training		94	21.1%
University Degree		274	61.6%
Other		28	6.3%
**Employment Status**		**452**	
Full-time Employment		156	34.5%
Part-time Employment		82	18.1%
Stay-at-home parent/unemployed		214	47.3%
**Marital Status**		**456**	
Married		318	69.7%
Domestic Partnership		33	7.2%
Never married/Separated/Divorced/Widowed		105	23.0%
**Location**		**454**	
Urban Setting		194	42.7%
Suburban Setting		161	35.5%
Rural Setting		90	19.8%
Remote Setting		9	2.0%
**Type of Child’s Diagnosis**		**456**	
Autism Spectrum Disorders		193	42.3%
Attention Deficit Hyperactivity Disorder		170	37.3%
Intellectual Disability		112	24.6%
Global Developmental Delay		103	22.6%
Learning Disability		89	19.5%
Cerebral Palsy		72	15.8%
Epilepsy		70	15.4%
Fetal Alcohol Spectrum Disorder		32	7.0%
Down Syndrome		13	2.9%
Spina Bifida		2	0.4%
Other^a^		163	35.7%
	*M*	*SD*	*range*
Age of Parents (in years)	43.12	7.58	24–69
Age of Child (in years)^b^	11.63	5.94	2–42

^a^Example of other diagnoses included Chromosome 18 duplication syndrome, Rett syndrome, and neurofibromatosis

^b,c^This information was reported based on the participant’s child with IDD. If they had more than one child with IDD, they were asked to report the conditions for the child with the most severe challenges. In the case of equal challenges, they answered based on their oldest child with IDD

A generally high prevalence of barriers was observed (*M* = 20.93, *SD* = 9.28); especially on taking caregiving as a priority (i.e., participants perceived this priority affected seeking help for their own mental health challenges) (93.4%), not having enough time (90.4%), and high costs (85.8%). The least experienced barriers were discouragement from people around (18.8%), confidentiality concerns (29.1%), and the fear of losing control/autonomy in a treatment (36.2%); see Table 2 for more information.

**Table 2:** Frequency of the PHBS by Items

Item	*M*	*SD*	Not at all	A little bit^1^	Moderately to Extremely^a^
(1) I don’t have enough time	2.54	1.34	9.6%	16.4%	74.0%
(2) Support is too far away	1.52	1.38	33.8%	18.8%	47.4%
(3) The expense and added costs (e.g., time off work, transportation) are too high	2.44	1.44	14.2%	15.8%	70.0%
(4) I don’t have access to support that is based on the latest research.	1.35	1.42	41.6%	18.6%	39.8%
(5) I don’t know how to get access to support.	1.2	1.32	43.9%	19.7%	36.4%
(6) The waiting lists are too long	2.01	1.56	25.9%	15.6%	58.5%
(7) I am not emotionally ready for receiving support	0.79	1.05	53.0%	26.3%	20.7%
(8) It might not be confidential	0.56	1.06	70.9%	14.5%	14.6%
(9) Support would not be helpful for me	0.62	1.04	66.3%	16.4%	17.3%
(10) Support involves loss of control/autonomy	0.58	0.93	63.8%	21.9%	14.3%
(11) I don’t want to be labelled as having a mental illness	0.76	1.2	61.9%	18.8%	19.3%
(12) I feel guilty for having mental health challenges from caring for my child.	1.5	1.46	36.9%	19.2%	43.9%
(13) My child and my family are my priority; I have to focus on caregiving	2.75	1.3	6.6%	15.1%	78.3%
(14) The people around me discourage me from seeking help for mental health challenges	0.3	0.72	81.2%	10.9%	7.9%
(15) I want to avoid talking about stressful experiences in my life.	1.27	1.24	34.6%	29.9%	35.5%
(16) Other^b^	0.68	1.33	76.4%	3.2%	20.4%

^a^For the participants who endorsed the barrier, they rated to what extent the barrier affected their access to mental healthcare

^b^For the open-ended question “please specify other barriers”, forty effective answers were collected. The most common barriers the participants reported were no caregiver-specific treatments for them (*n* = 13/40, 32.5%) and financial issues (*n* = 7/40, 17.5%)

### Item reduction and dimensionality

In the scale development phase of the PHBS, item reduction and extraction of factors were employed. The item reduction technique involves item discrimination tests and item-total correlations. As shown in the Table 3, all items showed good discrimination and moderate to high correlations with the scale total scores. Among all items, item 8 (confidentiality concern), item 5 (no access to healthcare support), and item 15 (parents’ own avoidance) revealed the highest item-scale correlations with the total barriers parents experienced or perceived.

**Table 3:** Item Discrimination Index and Item-Total Correlation for the PHBS

Item	Upper Group	Lower Group	Discrimination Index	***r***_**pm**_
Item 1	.974	.770	.204	.395**
Item 2	.805	.390	.415	.471**
Item 3	.941	.630	.311	.478**
Item 4	.874	.270	.604	.551**
Item 5	.856	.170	.686	.562**
Item 6	.944	.412	.532	.512**
Item 7	.703	.130	.573	.465**
Item 8	.655	.070	.585	.586**
Item 9	.483	.160	.323	.264**
Item 10	.602	.110	.492	.505**
Item 11	.632	.170	.462	.486**
Item 12	.890	.320	.570	.545**
Item 13	.992	.828	.164	.487**
Item 14	.385	.040	.345	.371**
Item 15	.907	.390	.517	.560**
Item 16	.403	.141	.262	.298**

Discrimination Index (*I*) = *P*_*(U)*_ – *P*_*(L)*_, where *P*_*(U)*_ is the proportion of cases in the upper group and *P*_*(L)*_ is the proportion in the lower group, using the 25th percentile and 75th percentile total score as cutoff scores. *r*_pm_ refers to the Pearson product-moment correlation coefficients between the item and the PHBS total score. ** indicates *p* < .01

A principal component analysis (PCA) was run on the 15-question PHBS scale on 456 parent participants. The PCA revealed four components that had eigenvalues greater than one and which explained 24.72, 12.45, 9.25, and 7.76% of the total variance, respectively. Visual inspection of the scree plot indicates the four components should be retained. The four components explained 54.17% of the total variance. A direct Oblimin oblique rotation was employed to aid interpretability because correlational relationship between components were observed. The interpretation of the data was consistent with the attributes that the questionnaire was designed to measure, with personal belief items on component 1, support accessibility items on component 2, resource availability on component 3, and emotional readiness on component 4. There were no hyperplane items (items with loadings on no factor). Items 2, 3, 8, and 15 had salient loadings on more than one factor (see Table 4 for details). The component that an item belongs to depends on the magnitude of factor loadings and the concept that the item content conceptually overlaps with. All four components had sufficient items (item *n* > 3). All communalities were strong (communalities = [.433, .653]). Note that item 8 (“It might not be confidential”) was classified in component 1, although it had higher loading in component 2, for two reasons: (1) the item conceptually overlapped with personal belief more than with support accessibility; and (2) the loading in component 1 was acceptable (component coefficient = 0.436 for component 1 and 0.537 for component 2). Four factor scores were calculated and entered a second PCA to assess whether the components converged into a single factor (i.e., barriers to accessing care). The second PCA confirmed a single attribute (eigenvalue = 1.89); therefore, the use of a single total score to interpret perceived barriers is supported. Component loadings and communalities of the rotated solution are presented in Table 4.

**Table 4:** Rotated Structure Matrix for PCA with Varimax Rotation of a Four Component PHBS

Item	Rotated Component Coefficients				
	Component 1	Component 2	Component 3	Component 4	Communalities
Item 12	**0.760**	0.188	0.271	0.082	.639
Item 11	**0.696**	0.161	−0.065	0.409	.553
Item 14	**0.577**	0.266	−0.202	0.206	.433
Item 15	**0.573**	0.183	0.22	0.485	.473
Item 8	**0.436**	0.537	0.06	0.414	.472
Item 4	0.094	**0.752**	0.131	0.192	.580
Item 6	0.136	**0.748**	0.106	0.03	.566
Item 5	0.300	**0.746**	0.111	0.048	.590
Item 1	0.108	0.043	**0.798**	0.112	.653
Item 3	0.066	0.412	**0.629**	0.007	.501
Item 2	−0.024	0.496	**0.526**	0.106	.478
Item 13	0.503	0.076	**0.512**	0.137	.486
Item 10	0.359	0.227	−0.001	**0.764**	.621
Item 9	0.016	−0.026	−0.024	**0.733**	.584
Item 7	0.282	0.154	0.171	**0.684**	.496

Factor loadings in the component they were classified into are highlighted in bold

### Reliability, concurrent validity, and discriminant validity

The reliability was evaluated by internal consistency. Cronbach’s α was .77 for the PHBS whole scale, .67 for component 1 (i.e., personal belief), .69 for component 2 (support accessibility), .57 for component 3 (resource availability), and .60 for component 4 (emotional readiness). The concurrent validity of the PHBS was evaluated by its correlation with physical health, mental health, and social support. There were statistically significant, moderate, positive correlations between barriers and the parental well-being (physical health, *r* (453) = .276, *p* < .0005; mental health, *r* (453) = .325, *p* < .0005). This means a higher level of barriers in receiving mental healthcare was associated with generally poorer health status. A statistically significant, moderate negative correlation was observed between PHBS and social support scores, *r* (451) = .273, *p* < .0005. This means higher barriers in seeking support were associated with lower perceived social support.

Discriminant validity was tested by its correlational relationship with parenting. There was an insignificant and weak correlational relationship between barriers and parenting, *r* (391) = .092, *p* = .070, indicating there was no reliable or strong correlation between parents’ experiencing of barriers in seeking support and their parenting styles (see Table 5 for details).

**Table 5:** Correlations between the PHBS and Other Selected Scales

Variable	Global Physical Health	Global Mental Health	Social Support	Parenting
Parental Healthcare Barriers Scale (PHBS)	.26**	.32**	−.29**	.10
	[.17, .35]	[.23, .40]	[−.37, −.20]	[−.01, .20]
PHBS - support accessibility	.30**	.26**	−.21**	.09
	[.21, .38]	[.17, .34]	[−.30, −.12]	[−.02, .19]
PHBS - personal belief	.23**	.30**	−.28**	.14**
	[.14, .31]	[.21, .38]	[−.37, −.19]	[.04, .24]
PHBS - emotional readiness	.04	.03	−.07	−.02
	[−.05, .14]	[−.06, .13]	[−.17, .02]	[−.12, .08]
PHBS - resource availability	.15**	.25**	−.16**	.03
	[.05, .24]	[.16, .33]	[−.25, −.07]	[−.08, .13]

Values in square brackets indicate the 95% confidence interval for each correlation. * indicates *p* < .05. ** indicates *p* < .01

The three subscales of the PHBS (i.e., support accessibility, personal belief, and resource availability) also showed weak to moderate positive correlations with global mental and physical health challenges (*r* (453) = [.16, .32], *p* < .05) and moderate negative correlation with social support (*r* (451) = [−.27, −.15], *p* < .01). The emotional readiness subscale did not reveal significant correlations with global mental health (*r* (453) = .05, *p* = .13), physical health (*r* (453) = .05, *p* = .32) or social support (*r* (451) = −.07, *p* = .13).

### Barriers and geographic settings

The total sum score of barriers increased from suburban group (*M* = 20.56, *SD* = 9.35), to urban group (*M* = 20.78, *SD* = 9.03), to rural and remote group (*M* = 21.53, *SD* = 9.49); however, the differences between these geographic groups were not statistically significant, *F* (2, 451) = .356, *p* = .701. The three groups did not reveal statistically significant differences in 3 of the 4 subscales either: support accessibility barriers, *F* (2, 451) = 1.059, *p* = .348, personal belief barriers, *F* (2, 451) = 1.665, *p* = .190, or emotional readiness barriers, *F* (2, 451) = .367, *p* = .693. The resource availability barrier was statistically significantly different between different geographic groups *F* (2, 451) = 3.643, *p* < .05, *η*^*2*^ = .016. The barriers regarding resource availability increased from the urban group (*M* = 2.22, *SD* = 0.87), to suburban group (*M* = 2.27, *SD* = 0.93), to rural/remote group (*M* = 2.51, *SD* = 0.90). Tukey post hoc analysis revealed that the mean increase from rural/remote to suburban group (0.24, 95% CI [− 0.03, 0.51]) was marginally statistically significant, *p* = .091, and the increase from rural/remote to urban group (0.29, 95% CI [0.03, 0.55]) was statistically significant, *p* = .023, but not statistically significant between suburban and urban group.

## Discussion

The current study systematically detected and classified the barriers that parents of children with IDD reported when seeking mental health treatments for themselves. The investigation was performed with extensive literature search, relatively rigorous scale development process, and a national and broad sample. The predominant barriers these parents had were connected to prioritizing their role as a caregiver (93.4%) and that they did not have enough time for their own health challenges (90.4%). The major barriers found in our study are in line with previous research results among parents of children with disabilities [11, 12]. The responsibility of caring for their children with IDD hindered the parents’ motivation and resources to deal with personal mental health problems. The impact of caring for children with IDD on parents’ personal life was also presented with the relative high education level (e.g., over 60% received university degrees) and only a half of employment rate.

Some barriers found in our study have also been reported in prior literature, such as negative personal beliefs about the services [25], insufficient treatment resources [14], and high service costs [15]. Compared to the samples from previous studies, our sample perceived higher levels of barriers, with 57.0% reporting high service costs, 8.5% reporting long waitlists, and 39.8% reporting lack of access to treatment. These differences can potentially be explained by differences in healthcare accessibility in other countries (Paula et al., 2020). Moreover, costs for healthcare services in Canada are high in comparison to some other countries [61]. Moreover, our study found the barriers that were not widely discussed (e.g., no caregiver-specific treatments for parents of children with IDD); which could be added and assessed as an additional item for a revised PHBS later on.

The PHBS showed sound psychometric properties and contains four dimensions of barriers to accessing mental healthcare: *support accessibility*, *personal belief*, *emotional readiness*, and *resource availability*. The total scale and three of the four subscales (i.e., support accessibility, personal belief, and resource availability) also showed good construct validity, with moderate positive correlations with mental and physical health challenges and a moderate negative correlation with social support.

This study compared barriers perceived by Canadian parents in different geographic settings. There was no significant difference of barriers in rural/remote, urban, or suburban groups, but rural and remote groups perceived significantly more barriers in resource availability. This confirmed the mental healthcare inequity found in the general population [23, 24]. The finding also implies that it is key to address parents’ encountered barriers to access mental healthcare in rural and remote areas and to increase equitable resource allocation.

The results of this study have various practical implications. These include: (1) to improve financial support for parents of children with IDD who need mental health services, (2) to deliver time-flexible (e.g., asynchronous) or time-efficient interventions to accommodate parents’ priority of caregiving; and (3) to increase service availability by providing more accessible evidence-based interventions, such as e-health programs. Our study discovered that some parents might not feel emotionally ready for mental health treatments. Providing different types of mental health treatments with different degrees of intensity could encourage parents with some doubts to start on a low-investment program. This could include designing goal-oriented, motivation and engagement targeted, and person-centred mental healthcare [62]. The identification of these obstacles could reduce the information gap between care deliverers and support seekers.

This study has some limitations: the current study only included parents in Canada and parents of children with IDD. Parents in other countries may face other challenges in terms of mental health services, such as a lack of infrastructure and fragmented and inefficient collaboration in the mental healthcare system [63]. The distribution of barriers may not be further generalizable to parents of children with other disabilities, such as cancer survivors. Although the sample in the current study was nationwide and relatively broad, there is a need for multiple assessments in a variety of samples to evaluate its reliability. For example, this and other studies in the field [10, 13] recruited more female participants than male participants. This might imply that mothers are more likely to be main caregivers of children with IDD. Efforts should be made to retest the scale in a gender-balanced sample. This is also essential to confirm the four-factor model of PHBS, which was only initially validated in the current study. Finally, due to the pandemic, the study was conducted entirely online and the access of the survey was restricted to parents with access to the Internet. This may have biased the results of the named barriers, especially the barriers with respect to resource availability. It might manifest on the comparision of barriers in different geographic settings as in this study less parents were recruited in rural and remote areas than those from urban and suburban areas.

In conclusion, the study illustrates that parents of children with IDD experienced various barriers when seeking mental helath services. The PHBS scale shows a good reliability and validity and evaluats parents’ barriers in four dimensions: support accessibility, personal belief, emotional readiness, and resource availability. Parents in rural and remote areas were likely to have more limited resources. Implications of the study include reinforcing time-flexible mental health interventions, improving financial support, and increasing service availability, and promoting equitable healthcare access.

## Supplementary Information

**Additional file 1. **Parental Healthcare Barriers Scale (PHBS).

## Data Availability

The datasets used and/or analysed during the current study are available from the corresponding author on reasonable request and with permission of IWK Health centre Research Ethics Board.

## Abbreviations

IDD: Intellectual and developmental disabilities
PCA: Principal components analysis
PHBS: Parental Healthcare Barriers Scale
