# Supplementary material for: How to measure barriers in accessing mental healthcare? Psychometric evaluation of a screening tool in parents of children with intellectual and developmental disabilities
Source: BMC Health Serv Res. 2022 Nov 21;22:1383. doi: 10.1186/s12913-022-08762-0 (PMC9677628; doi:10.1186/s12913-022-08762-0)
Supplement: Supplementary file 1 — Additional file 1. Parental Healthcare Barriers Scale (PHBS). [file 12913_2022_8762_MOESM1_ESM.docx]

**Additional File 1 - Parental Healthcare Barriers Scale (PHBS)**

When looking or receiving support for mental health challenges related to caregiving, several obstacles can get in your way.

| **Please indicate to what extent the following statements affected your access to care?** | Not at all | A little bit | Moderately | Quite a bit | Extremely |
| --- | --- | --- | --- | --- | --- |
| (1) I don’t have enough time |  |  |  |  |  |
| (2) Support is too far away |  |  |  |  |  |
| (3) The expense and added costs (e.g., time off work, transportation) are too high |  |  |  |  |  |
| (4) I don’t have access to support that is based on the latest research. |  |  |  |  |  |
| (5) I don’t know how to get access to support. |  |  |  |  |  |
| (6) The waiting lists are too long |  |  |  |  |  |
| (7) I am not emotionally ready for receiving support |  |  |  |  |  |
| (8) It might not be confidential |  |  |  |  |  |
| (9) Support would not be helpful for me |  |  |  |  |  |
| (10) Support involves loss of control/autonomy |  |  |  |  |  |
| (11) I don’t want to be labelled as having a mental illness |  |  |  |  |  |
| (12) I feel guilty for having mental health challenges from caring for my child |  |  |  |  |  |
| (13) My child and my family are my priority; I have to focus on caregiving |  |  |  |  |  |
| (14) The people around me discourage me from seeking help for mental health challenges |  |  |  |  |  |
| (15) I want to avoid talking about stressful experiences in my life |  |  |  |  |  |
| (16) Other, please specify: _____ |  |  |  |  |  |
